# Validation of a Wireless Catheter-Free Ambulatory Urodynamics Device in Women With Neurogenic Bladder

**DOI:** 10.1002/nau.70172

**Published:** 2025-11-06

**Authors:** Michael D. Gross, Brendan T. Frainey, Madison E. Lyon, Kevin C. Lewis, Mohamed Elazab, Hassaan A. Bukhari, Tyler Tevis, Robert S. Butler, Reilly Burhanna, Cole Smith, Howard B. Goldman, Ly Hoang-Roberts, Margot S. Damaser, Steve J. A. Majerus

**Affiliations:** 1 Glickman Urology Institute, Cleveland Clinic, Cleveland, Ohio, USA; 2 Department of Biomedical Engineering, Cleveland Clinic Research, Cleveland, Ohio, USA; 3 Advanced Platform Technology Center, Louis Stokes VA Medical Center, Cleveland, Ohio, USA; 4 Quantitative Health Sciences Department, Cleveland Clinic, Cleveland, Ohio, USA; 5 Department of Electrical, Computer, and Systems Engineering, Case Western Reserve University, Cleveland, Ohio, USA

**Keywords:** ambulatory urodynamics, female, multiple sclerosis, telemetric ambulatory urodynamic monitoring, urodynamics

## Abstract

**Aims:**

Urodynamics (UDS) is critical for patients with neurogenic bladder but remains artificial given retrograde filling and voiding around a catheter in an uncomfortable setting. We have developed a wireless device for catheter-free real-time measurement of intravesical pressure during natural filling. Women with neurogenic bladder secondary to multiple sclerosis (MS) may experience discomfort, detrusor overactivity, or inability to urinate while observed during UDS which might belie true voiding patterns. The aim of this study was to test the wireless device in women with neurogenic bladder due to MS.

**Methods:**

Ten female participants with neurogenic bladder secondary to MS underwent standard UDS, after which the device was inserted and a second UDS study performed. Patients then ambulated with only the device in place for an additional void.

**Results:**

There were no significant differences in UDS results, pain or discomfort between the first and second cycle. The device captured 98% of UDS events, including 100% of detrusor overactivity. Post void residual volume after UDS (160 ± 179 mL [Range 0–454]) was significantly greater than after ambulation with the wireless device alone (19 ± 18 mL [Range 0–46]; *p* = 0.01), demonstrating greater voiding efficiency with the wireless device alone than with the UDS catheter in place.

**Conclusions:**

The device was well tolerated without complications, captured urodynamic data with a high degree of fidelity, and demonstrated additional utility in patients with borderline obstruction or inability to urinate while observed who cannot void during standard UDS. This device offers a promising alternative to the critical information provided by UDS in a less-invasive, more physiologic manner.

## 1 ∣ Introduction

Urodynamics (UDS) is the gold-standard for the evaluation and monitoring of myriad bladder pathologies. This exam may produce significant physical and emotional patient discomfort and may misrepresent symptoms due to non-physiologic filling and voiding [1]. UDS is particularly crucial for patients with neurogenic bladder. Intravesical pressure monitoring can identify patients at risk of renal deterioration and inform treatment decisions [2]. Up to 90% of patients with multiple sclerosis experience bladder symptoms during their lifetime. These patients may void at lower pressures and over half exhibit detrusor overactivity (DO) [3]. In addition, these patients can experience detrusor-sphincter dyssynergia and have difficulty voiding, particularly around a urethral catheter as is required during UDS [3].

To address these limitations, our group has developed a novel wireless, catheter-free urodynamics monitor (UM) for the detection of intravesical pressure. The UM is inserted transurethrally into the bladder lumen, where it resides and transmits bladder pressure continuously to an externally worn radio and antenna system, which record the transmitted data and share it via Bluetooth with a nearby computer [4]. We previously demonstrated that this device is safe and accurate in a population of healthy women with symptoms of overactive bladder [5].

The aim of this study was to compare UM data during UDS and natural filling to conventional UDS in women with neurogenic bladder due to MS. We hypothesized that these subjects would have decreased postvoid residual volume (PVR) when voiding with the UM compared to UDS and that the UM will be safe, well tolerated, and provide accurate detection of intravesical pressure and bladder events.

## 2 ∣ Materials and Methods

This study was approved by the Cleveland Clinic Institutional Review Board (Study Number 20-1002). All participants gave informed consent for the study procedures. Participants were recruited from March 2021 to July 2023. Female patients over 18 years of age presenting to the urology office with lower urinary tract symptoms with a confirmed diagnosis of multiple sclerosis were identified for inclusion in the study if standard UDS was indicated. Patients were excluded if they could not ambulate or had an active MS flare, active urinary tract infection, stage 2B or worse pelvic organ prolapse, a concomitant diagnosis of interstitial cystitis or a history of radical pelvic surgery.

### 2.1 ∣ Urodynamics Monitor (UM)

**FIGURE 1 ∣:**
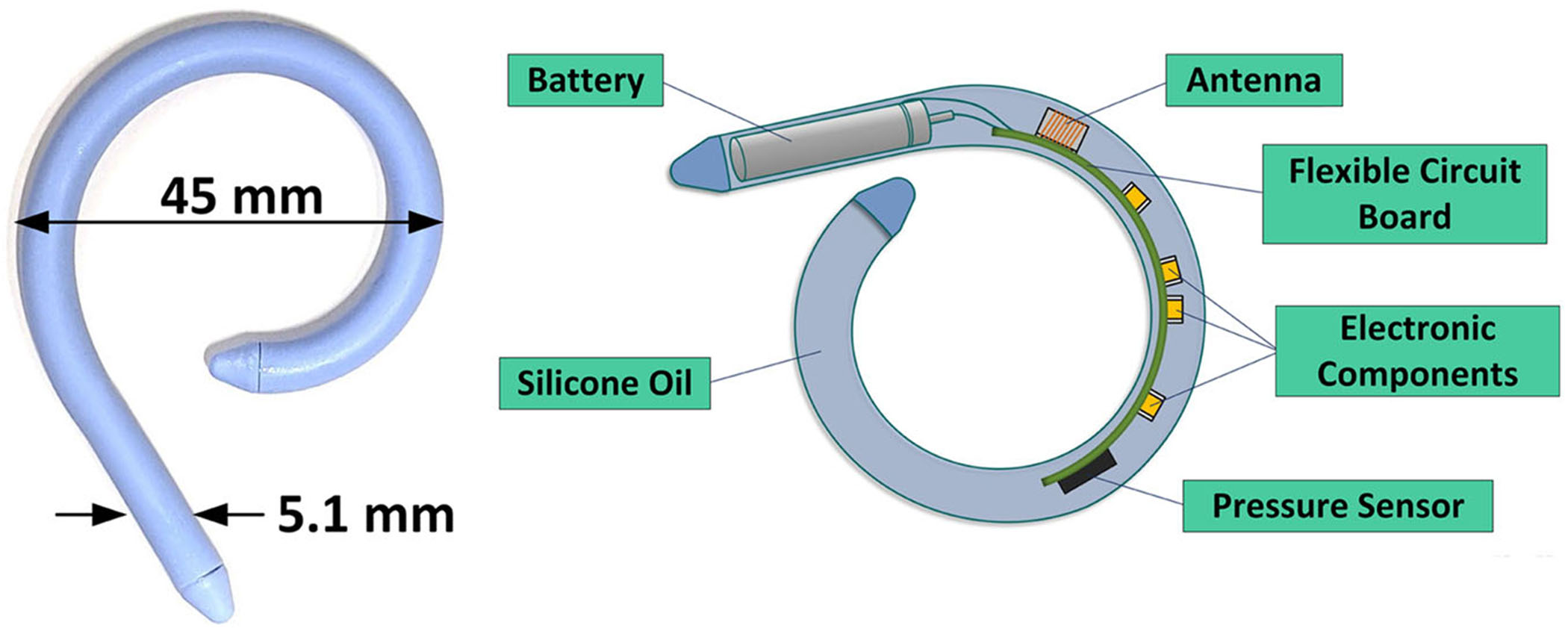
The Urodynamics Monitor measures approximately 5.1 mm (15.3 Fr) and includes internal flexible, wireless pressure-sensing electronics. The housing is made from medical-grade silicone filled with medical-grade silicone oil to enable internal pressure measurement.

The UM consists of a custom, flexible printed circuit board, a microcontroller, a data transmitting antenna, a pressure sensor, and a lithium-ion pin battery (Figure 1). Custom software was programmed onto the microcontroller which managed device power consumption and handled radio communication of pressure data. The flexible electronics module was enclosed in a custom designed medical-grade silicone housing which was filled with silicone oil, reducing signal drift compared to aqueous suspension [6]. Compared to the device used in a previous study [5], in this study the UM used a softer silicone housing and a different silicone oil due to supply chain sourcing challenges. The UM was sterilized via ethylene oxide gas sterilization and outgassed for at least 24 h before patient use. The UM device was calibrated in a container of sterile saline solution before insertion by measuring the height of the water column to calculate the pressure head.

Once activated, the UM has sufficient battery life to transmit pressure data 10 times per second (10 Hz) for 135 h at a distance of about 50 cm in laboratory conditions. Data are wirelessly transmitted from the UM to a 9-cm diameter loop antenna taped to the lower abdominal skin. The antenna is connected to a pager-like portable radio which records data on a microSD card and further transmits data via Bluetooth to a PC for real-time viewing. A sterile 0-silk or 2-0 monofilament suture is threaded through one endcap of the UM to enable transurethral extraction. Details of UM manufacture and use have been previously published [4, 5].

### 2.2 ∣ Experimental Procedure

A pre-procedure urine culture was obtained and any positive culture with a uropathogen was treated before the testing. Patients first performed uroflowmetry. The post-void residual volume (PVR) was then determined by measuring the volume of urine drained after placement of the vesical urodynamics catheter.

All patients had baseline multi-channel UDS, including cystometry, pressure-flow studies, and electromyography using an Aquarius CT urodynamics machine (Laborie Medical Technologies, Ontario, Canada) using UDS 120 software version 14 (Laborie Medical Technologies). UDS was performed using a 7.4 French water-filled catheter (catalog number 020707-B, Cook Medical Europe Ltd., Limerick, Ireland) for intravesical pressure measurement (Pves) and a 9 French water-filled catheter (Laborie Medical Technologies, catalog number RPC-9) for intraabdominal pressure measurement (Pabd). UDS parameters were recorded for all study participants per ICS standards [7].

After standard UDS was completed, the vesical catheter was removed. PVR was then determined as the difference between the infused and voided volume during the study. The UM was then inserted per urethra followed by replacement of a vesical catheter. A second cycle of UDS was then performed while recording simultaneously from the UM. PVR was again measured as the difference between infused and voided volume. This protocol follows that described by Frainey et al., except that post-UM insertion cystoscopy was not performed in the current study [5]. The vesical and rectal catheters were then removed and the patient ambulated around the clinic, filling naturally while the UM system recorded vesical pressure, until they voided at least one additional time, this time privately on a toilet in the bathroom with the door closed. Afterward, an EchoNous portable bladder ultrasound was used to determine PVR. If a patient voided multiple times, only the first void was considered for statistical analysis. The transurethral suture was then used to remove the device.

Patients were surveyed for pain via a visual analog scale before the procedure, at the end of the testing session, and at 48 h posttest. Patients were additionally assessed for their comfort level at each discrete stage of the procedure on a 5-point Likert scale including each individual round of UDS, device insertion, removal, and the ambulatory portion of the study. A post-procedure urine culture and heavy metal screen were obtained.

### 2.3 ∣ Data Analysis

Urodynamic pressure data were extracted from clinical machines but only the Pves channel was used for comparison against UM data. Data were processed in MATLAB (MathWorks, Nattick, MA, USA, R2023b version). Urodynamic Pves and UM-measured vesical pressure waveforms were time-aligned using a patient cough for synchronization. UM data were converted to calibrated units of pressure using a two-point linear calibration obtained in a water chamber before patient use. Additional patient-dependent offset was removed as referenced to the baseline (empty) pressure obtained from Pves. Outliers were removed from both data sources using a length-100 Hampel filter with a threshold of seven standard deviations. While the UM records pressure at 75 Hz, it applies a 0.5-Hz low-pass filter before data transmission. Therefore, UDS data were also low-pass filtered to 0.5 Hz bandwidth using an 8th-order digital filter.

Since data were collected on separate systems with slightly different clock timing, time alignment was performed to synchronize simultaneous UDS Pves data with UM pressure data. Following alignment, a narrow range of conversion factor adjustments was applied to minimize artifacts. Regions of interest were then selected based on segments that were valid in both the UDS and UM datasets (i.e., catheter in the bladder for Pves data and no dropouts in UM data). A nonlinear high-pass median filter (window length of 500 s, resulting in a corner frequency of 0.001 Hz) was subsequently implemented [5] to remove baseline drift. Linear regression analysis was performed to assess the correlation between UM and UDS pressure values. A Bland-Altman plot was generated to evaluate agreement between simultaneous UM and UDS pressure measurements and to identify any systematic bias.

Urodynamic parameters and pain/discomfort scores are presented as median and range. Patient specific variables including age, BMI and PVR are presented as mean ± standard deviation and range. Urodynamic exams were reviewed independently by two authors who then met to resolve conflicts. Abdominal events were distinguished from detrusor overactivity by their morphology when interpreting the UM-measured data. Pabd and Pdet were used to confirm the characterization of abdominal events and detrusor overactivity on standard UDS for comparison. Study notes indicating permission to void distinguished volitional voiding from other events. Given different lengths of the observation period between UDS and the ambulatory phase of the protocol, the incidence of detrusor overactivity per patient was calculated per unit time of observation (i.e., events per minute). A repeated measures ANOVA was used to compare results from repeated urodynamic exams, frequency of DO, and pain and tolerability scores with the Dunnett-Hsu adjustment for multiple comparisons. PVR at each phase of the procedure were compared with repeated measures ANOVA with Dunnet-Hsu adjustment for multiple comparisons. In all cases, *p* < 0.05 indicated a statistically significant difference between groups.

## 3 ∣ Results

Eleven female patients consented for participation of whom 10 underwent study testing. One patient never reported for her testing appointment and was lost to follow up. Mean patient age was 50.8 ± 13.7 years (Range 21.7–71.5 years) and mean BMI was 26.8 ± 6.0 kg/m^2^ (Range 19.1–35.8 kg/m^2^). There was no significant change in UDS parameters after insertion of the UM including capacity, maximum flow rate, pressure at maximum flow rate and PVR (Table 1). The mean time to insert the device was 20 ± 6 s (Range 12–31 s) and removal took an average of 2 ± 1 s (Range 0.5–3.5 s). Patients ambulated with the UM in place for an average 52 ± 18 min (Range 20–84 min) and all patients were able to void at least once with the UM in situ. There were no post-procedural complications, urinary tract infections, or detectable heavy metals in the urine.

There were no significant differences in pre-procedure pain compared to pain immediately post procedure and pain 48 h postprocedure (Table 2). The median discomfort with UM insertion was 1.0 and with ambulation with the device alone was 0.5. There was no significant difference between the discomfort associated with any of the UM-specific portions of the procedure compared to the baseline UDS cycle.

**FIGURE 2 ∣:**
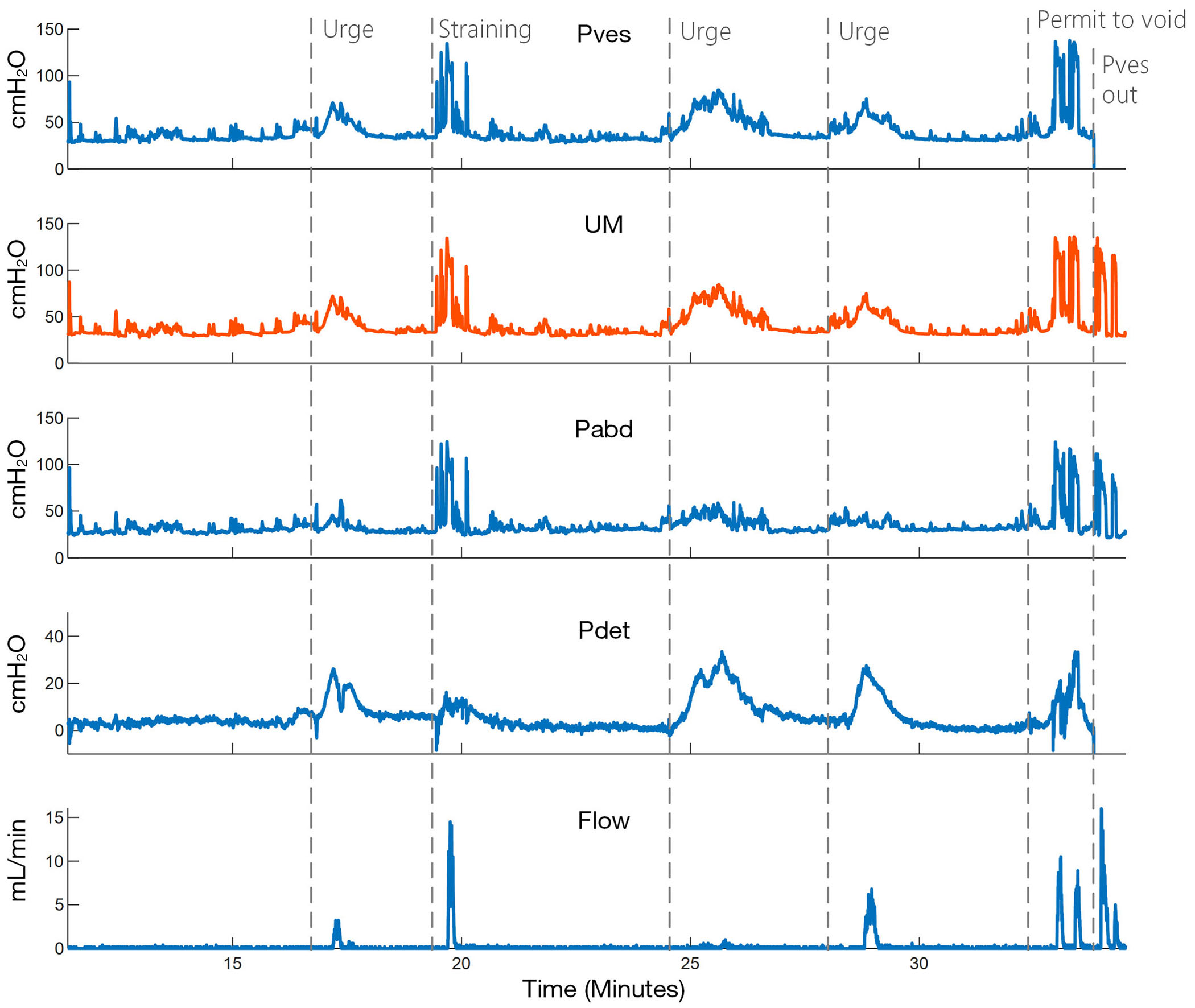
Intravesical pressure measured simultaneously via urodynamics catheter (Pves, top) and the wireless monitor (UM, 2nd from top) during one filling and voiding phase of urodynamics, abdominal pressure (Pabd, 3rd from top), detrusor pressure (Pdet 4th from top) and associated flow (bottom). Urodynamic events noted via dotted gray lines including removal of vesical catheter with continued recording by the wireless monitor with associated flow.

The UM detected 44/45 (98%) of urodynamic events as seen on the UDS tracing. The one missed event was during a period of signal dropout from wireless receiver antenna malposition. In one example subject’s data there were six total DO events in the first cycle of UDS, six events in the second cycle with simultaneous UM data collection (Figure 2) and two events during ambulation with the UM alone. Frequency of DO during baseline UDS (0.084 ± 0.141 DO/min [Range 0–0.39 DO/min]) was significantly greater than frequency of DO episodes during the ambulatory phase of the study (0.004 ± 0.008 DO/min [Range 0–0.02 DO/min]; *p* = 0.045) despite the longer period of time during which pressure was recorded during the ambulatory phase. The mean duration of the first phase of UDS (16.6 ± 5.7 min [Range 8–26 min]) was significantly shorter than the ambulatory phase (51.8 ± 17.6 min [Range 20–84 min]; *p* < 0.01). Frequency of DO did not differ between the first and second UDS.

**FIGURE 3 ∣:**
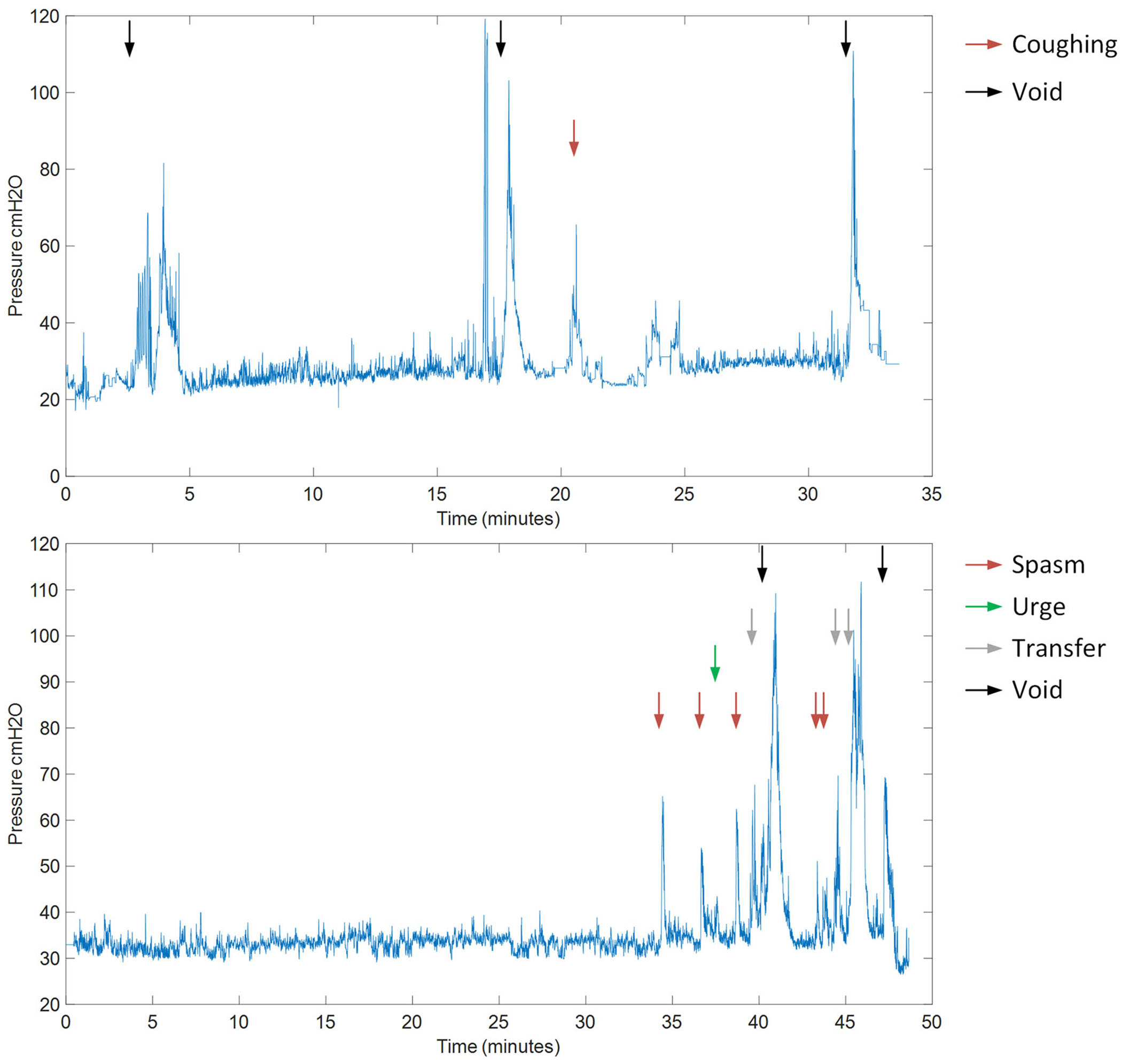
Ambulatory tracing recordings from wireless urodynamic monitor while patient walked around the clinic. Events noted. “Spasm” indicates leg spasm. Transfer indicated transfer between wheelchair and bed or toilet. Voiding occurred in a private bathroom in seated position. PVR with upper tracing = 0 mL, PVR with lower tracing = 29 mL.

Two subjects were unable to void during UDS. In these cases the vesical catheter was removed to enable voiding while the UM continued to transmit vesical pressure (Figure 2). An additional three subjects were only able to void small volumes during UDS but emptied almost completely during the catheter-free UM void. All subjects were able to spontaneously void during the ambulation phase of the study (Figure 3).

**FIGURE 4 ∣:**
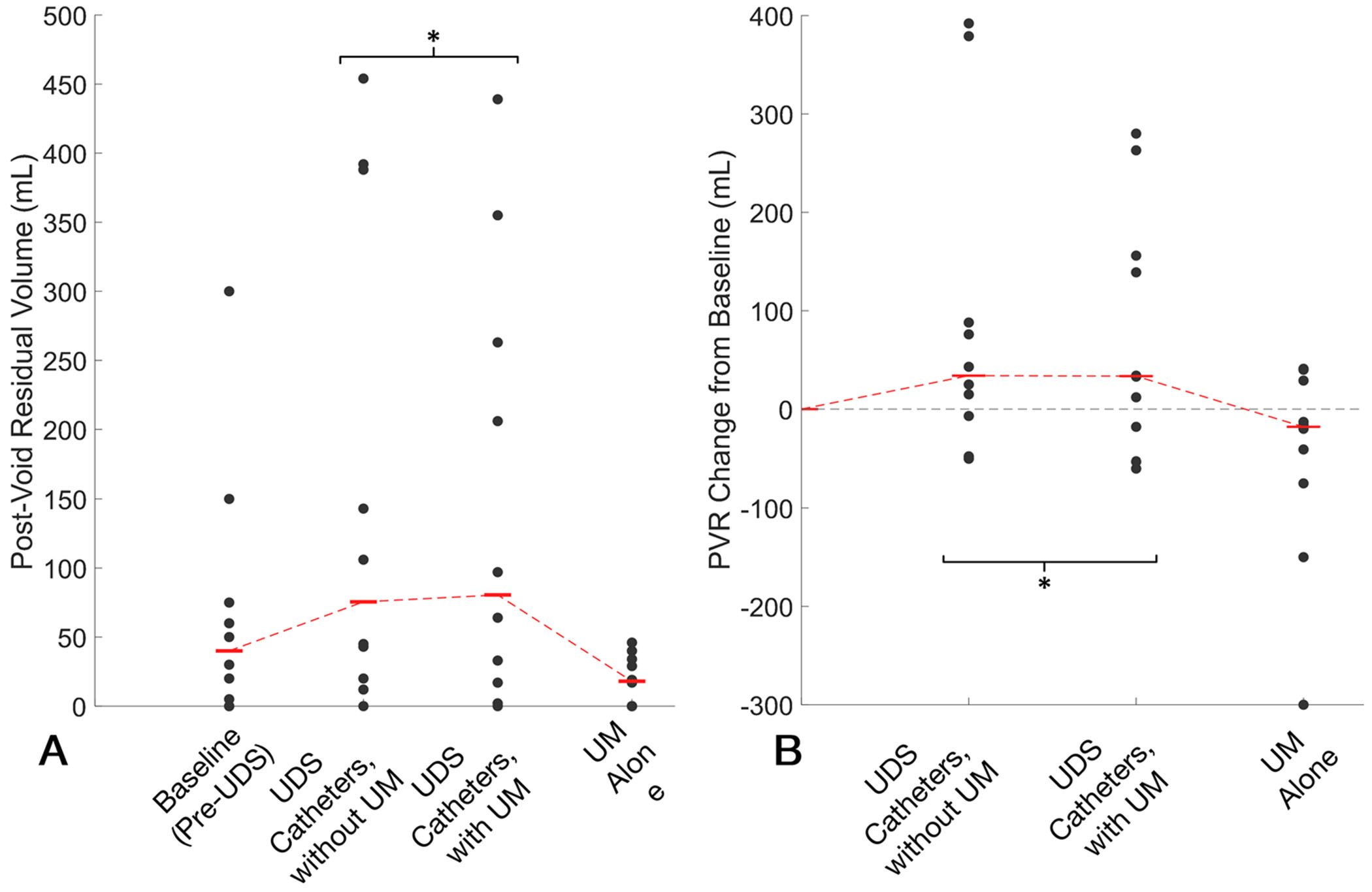
Post void residual urine volume (PVR) outcomes per patient measured before the procedure, during the first and second cycles of urodynamics and during ambulation with the urodynamics monitor alone (A) and with change in PVR per patient normalized to baseline PVR in different study phases (B). PVR was elevated relative to baseline when UDS catheters were in place (statistically significant changes indicated with *).

The PVR recordings exhibited large variability (Figure 4). The baseline PVR at presentation was 69 ± 93 mL (Range 0–300 mL). The mean PVR after the first UDS was 160 ± 179 mL (Range 0–454 mL) followed by 148 ± 159 mL (0–439 mL) on the second cycle and 19 ± 18 mL (Range 0–46 mL) during the ambulatory phase. Patients achieved a significantly lower PVR during the ambulatory phase compared to the first and second cycle of UDS (*p* = 0.01 and *p* = 0.02, respectively). Although the range was smaller, ambulatory PVR with the UM in place did not differ significantly from that obtained on baseline free flow (*p* = 0.55).

**FIGURE 5 ∣:**
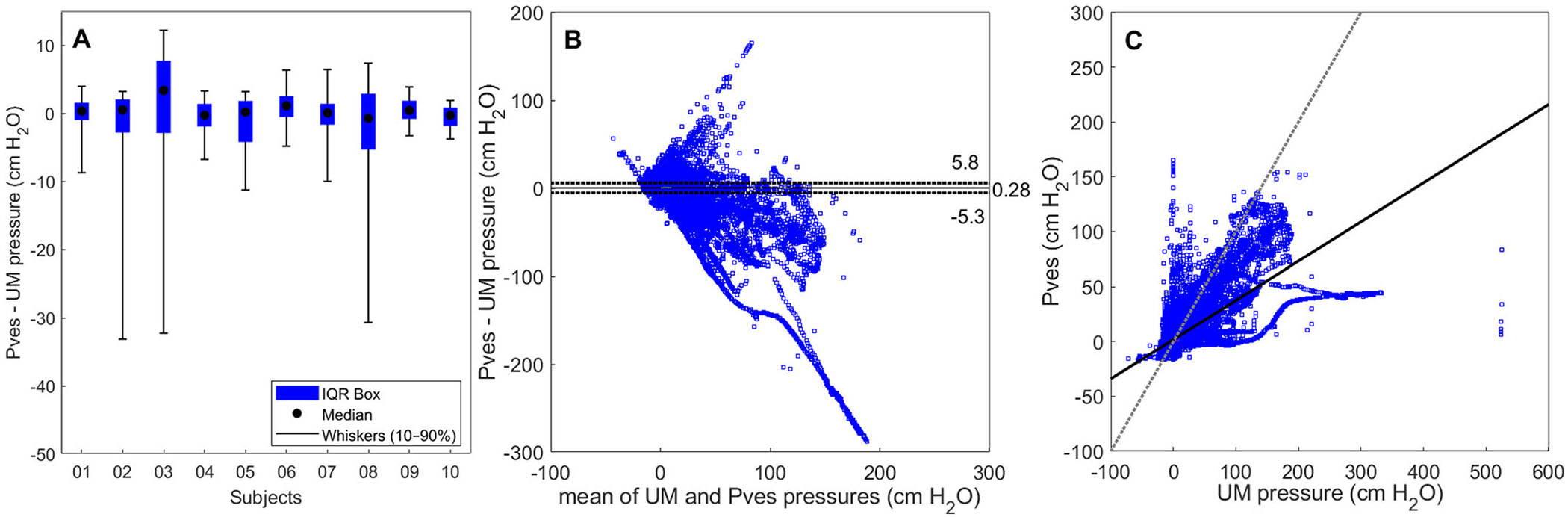
Graphical comparisons between urodynamic vesical pressure (Pves) and simultaneous Urodynamic Monitor (UM) pressures expressed as box plots of median and interquartile range (IQR) of Pves – UM pressure with whiskers indicating 10th and 90th percentile range (A). Bland-Altman plot comparing simultaneous Pves and UM recordings (B). The solid black horizontal line indicates the median difference, and the black dotted lines represent the median ± 1.45 × IQR. Linear regression of Pvs and simultaneous UM pressures (C). The dotted line represents the unity line. The solid line represents a linear fit with an R^2^ of 0.50.

Quantitative comparisons of pressures measured simultaneously with UDS catheters (Pves) and the UM demonstrated high agreement with variability between individual subjects. The narrow range of interquartile range demonstrates general agreement between the two measurement systems (Figure 5). Nonetheless, in three subjects, peak pressures measured with the UM were higher than pressure measured simultaneously with UDS catheters (Figure 5A). A Bland-Altman plot comparing simultaneous Pves and UM recordings showed a median bias (solid horizontal black line in Figure 5B) of 0.28 cm H_2_O with 1.45 × IQR (dotted horizontal black line in Figure 5B) from −5.3 to 5.8 cm H_2_O. Outliers were a small percentage of data with most pressure differences within 1.45 × IQR (77%–92%). Approximately 85% of the data points lie within the 1.45 x IQR limits, with fewer than 15% falling outside. Linear regression demonstrated a moderate correlation between the two measurement methods, with an R^2^ value of 0.50, indicating that 50% of the variance in UM pressure readings could be explained by UDS values.

## 4 ∣ Discussion

These data show that, despite some variability, the UM system gathered similar information in individuals with neurogenic bladder as standard UDS with only 2% reduction in the ability to detect clinically relevant events. The UM did not lead to earlier sensation or decreased capacity despite its presence in the bladder. The largest difference in clinical UDS outcomes was seen in increased pressure at peak flow rate with the UM present, although this difference was not statistically significant. Differences in the two UDS data may alternatively be attributed to the effect of repeated UDS which has been shown to demonstrate high variability [8]. Data reporting variability in pressure at peak flow is mixed, with one study in men reporting a significant decrease in maximum pressure after repeated UDS, contrasting with a study in 152 women showing no difference [9, 10]. Without subsequent differences in flow and voided volume, this effect, not seen in all patients, may not be clinically relevant.

We also did not observe a significant increase in detrusor overactivity events attributable to the device. In fact, during the ambulatory portion of the study, the incidence of non-voiding contractions was significantly less than during standard UDS. This may be due to the lower filling rate in natural filling than during UDS. In contrast, in a study by Radley et al. comparing conventional to ambulatory UDS, the incidence of DO was doubled during ambulatory testing with 59.5% of patients showing DO during ambulation after a previously negative cystometry [11]. It is worth noting that, in contrast to our study, these prior ambulatory UDS studies were done using catheter-based pressure measurements.

Jeon et al. also demonstrated a significantly increased rate of DO with UDS performed in the standing position compared to supine [8]. It has been debated whether this reflects provocation or improved symptom detection. In this regard, DO may be triggered by retrograde filling in even asymptomatic volunteers [12]. Taken together with a lack of increase in DO during the second UDS cycle and the excellent tolerability scores, one can infer that the UM device did not cause similar provocation of DO seen with standard catheters and retrograde filling. In fact, the UM thus may provide more accurate real-world data compared to traditional UDS.

Traditional pressure-flow UDS has a non-diagnostic rate of 19%–44% [1], partly attributable to patient anxiety and stress before and during testing, and potential obstruction by the catheter. Rosario et al. found 92.5% of patients with “bashful bladders” who could not void during video cystometry were able to generate voiding data on subsequent ambulatory UDS [13]. A similar study of 25 patients without any bladder contractility on standard UDS found evidence of contractility in 21 patients with ambulatory UDS [14]. The largest study to date showed 66 of 79 patients (84%) with suspected acontractility on UDS would have been misdiagnosed without the aid of ambulatory UDS [15]. Bashful bladder during UDS is otherwise understudied, but has been estimated to afflict up to 7% of the general population in public restrooms [16]. To reduce the incidence of this phenomenon, the International Continence Society Good Urodynamics Practice group recommends inquiry into patients’ baseline voiding anxiety, maximization of private voiding and finally, a specific diagnosis of inability to void on pressure flow studies in recognition of a nonrepresentative exam [7]. The privacy and minimal hardware afforded by the UM best realizes this recommendation by enabling patients to use a restroom as they normally would in private.

The presence of the UDS catheter can significantly alter voiding flow rate compared to free flow studies in multiple disease states [17]. This cannot be completely explained by the volumetric impact of the catheter and may additionally relate to insufficient sphincter relaxation in the presence of the catheter [18]. These inconsistencies logically begin to call established diagnostic criteria into question [19]. We saw this impact clearly in three patients who were only able to void small volumes during UDS but who emptied almost completely during the catheter-free UM void in this study. In two other subjects (one represented in Figure 2), additional voiding pressures during UDS were captured by the UM device after removal of the vesical catheter for inability to empty. In both phases of the study, we were able to measure vesical pressure data that is otherwise unobtainable during standard free flow while the subject voids seated and in private. While not statistically significant, the measured PVR was lower in the ambulatory phase than during the baseline uroflow. This may be attributable to increasing comfort with the testing center over time, voiding on a toilet as opposed to over the uroflow device, and potentially to the temporary impact of the prior fill and void cycles.

There are myriad advantages to obtaining ambulatory cystometric data. With prolonged monitoring, bladder events might be correlated to physical activity, dietary consumption, and diurnal variation. Ambulatory monitoring time during the current study is longer than previously published, making it the longest catheter-free testing period to date [5, 20]. Longer catheter-free recordings are feasible with the version of the UM used in this study (which can transmit for 135 h), but was not a focus in this phase of research. Nevertheless, the ability to capture long recordings of catheter-free bladder data promises large data sets for analysis which may require automated analysis method to facilitate interpretation. For example, our group has begun developing algorithms for the real-time classification of LUT events [21, 22]. Abdominal events may be further identified or filtered from pressure data alone or with the aid of an on-board accelerometer [22, 23]. Further study in this area will include monitoring at home and assessment of treatment efficacy through continued monitoring, as well as closed-loop or triggered neuromodulation [4, 24].

Quantitative comparison of simultaneous UDS Pves and UM pressure data demonstrated variability between subjects with occasional higher pressures measured by the UM. As a result, linear regression indicated a moderate correlation (R^2^ = 0.50), reflecting that while UM tracks pressure trends reasonably well, variability is likely due to insufficient quality control of laboratory manufactured UM devices, minor errors in temporal alignment of the two data sets, physiological differences, or measurement resolution. Nonetheless, Bland-Altman analysis demonstrated that 85% of the difference of simultaneous pressure data lies within 6 cm H_2_O (1.45 times the interquartile range), demonstrating sufficient agreement for clinical use, especially considering the catheter-free and wireless advantages of the UM.

The technology that forms the basis of the UM has been licensed to Bright Uro, who have received FDA approval for their product. Results of the initial study with this similar device have recently been published and demonstrate safety and tolerability of the Bright Uro system in a diverse Urologic patient population [20]. The results of the current study suggest that ambulatory bladder monitoring with a wireless catheter-free pressure measuring device could be a valuable adjunct to UDS assessment of LUT function.

This study had several limitations. There was inherent heterogeneity in the bladder symptomatology of patients with multiple sclerosis, however this supported the generalizability of presented results. Nonetheless, the small sample size warrants additional confirmatory studies. We did not insert urodynamics catheters during the ambulatory phase for correlation to the UM as our center does not have an established ambulatory UDS protocol, however this would have provided additional data for correlation and will be considered for future studies. The UM device measured only intravesical pressure and did not filter out abdominal events, however our group is refining algorithms to extract detrusor pressure from vesical pressure only [21, 22]. Alternatively, similar wireless monitoring devices have been proposed for use in the colon which could simultaneously provide abdominal pressure data [25]. The UM also did not measure volume, so it cannot replace all outcomes of UDS, although intravesical pressure-volume sensors have been recently demonstrated in large animals [26]. We did not measure bladder capacity in the ambulatory portion of the study so this is an area of future research.

The PVR in the ambulatory phase was measured via ultrasound rather than catheterization as performed in the other study phases, however the correlation coefficient of ultrasound measured PVR with catheterized PVR in experienced hands has been reported as high as 0.97 even with low volumes < 100cc [27]. Additionally, the PVR during UDS does not account for urine produced during testing, however with a mean testing duration of 16 min this is likely negligible. Finally, patients in the study were exclusively female owing to the ongoing work to refine an apparatus for insertion of the UM into the male urethra.

## 5 ∣ Conclusion

We demonstrated that the UM is safe and well tolerated in patients with neurogenic bladder due to multiple sclerosis. The UM also captures urodynamic events without affecting baseline urodynamic parameters. These data demonstrated additional value of the device beyond conventional UDS in the capture of vesical pressure during free-flow voiding and prolonged ambulatory monitoring without triggering additional symptoms. Wireless catheter-free bladder monitoring may be useful to provide real world vesical pressures over multiple fill and void cycles during ambulation and other activities.

## Figures and Tables

**TABLE 1 ∣:** Urodynamic measurements from UDS data with and without wireless urodynamic monitor (UM) in place.

Median (Range)	UDS Without UM	UDS With UM	*p* value
Capacity (cc)	326 (167–623)	323 (112–882)	0.894
First desire (cc)	144 (33–435)	167 (33–488)	0.126
Pdet at Qmax (cm H_2_O)	31.0 (14.6–63.5)	35.6 (6.5–97.5)	0.089
PVR (cc/s)	75.5 (0–454)	80.5 (0–439)	0.672
Qmax (cc/s)	11.6 (2.4–22.0)	13.8 (2.4–26.2)	0.848
Voided volume (cc)	157 (24–621)	186 (35–443)	0.988

Abbreviations: Pdet, Detrusor Pressure; PVR, Post void residual Volume; Qmax, Maximum Voiding Flow Rate.

**TABLE 2 ∣:** Pain and discomfort measures for study procedures.

Median (Range)	*n* = 10
Pain score [0–10]	
Baseline	1 (0–4)
Posttest	1 (0–5)
48 h posttest	0 (0–4)
Discomfort score [0–5]	
UDS cycle 1	2 (0–2)
UM insertion	1 (0–5)
UDS cycle 2	1 (0–2.5)
Ambulation with UM	0.5 (0–2)
UM removal	2 (0–4)

Abbreviations: UDS, urodynamics; UM, wireless urodynamic monitor.

## Data Availability

Individual patient data that underlie the results reported in this article, after deidentification, can be made available upon request.
